# ^1^H-NMR-Based Metabolic Profiling in Muscle and Liver Tissue of Juvenile Turbot (*Scophthalmus maximus*) Fed with Plant and Animal Protein Sources

**DOI:** 10.3390/metabo13050612

**Published:** 2023-04-28

**Authors:** Christina Hoerterer, Jessica Petereit, Gisela Lannig, Christian Bock, Bela H. Buck

**Affiliations:** 1 Alfred Wegener Institute for Polar and Marine Research, Biosciences, 27570 Bremerhaven, Germanybela.h.buck@awi.de (B.H.B.); 2 Faculty 1 Technology, University of Applied Sciences Bremerhaven, 27568 Bremerhaven, Germany

**Keywords:** insect meal, by-product, compound, glycogen, glucose, TMAO, betaine

## Abstract

Circular economy driven feed ingredients and emerging protein sources, such as insects and microbial meals, has the potential to partially replace fishmeal in diets of high-trophic fish. Even though growth and feed performance are often unaffected at low inclusion levels, the metabolic effects are unknown. This study examined the metabolic response of juvenile turbot (*Scophthalmus maximus*) to diets with graded fishmeal replacement with plant, animal, and emerging protein sources (PLANT, PAP, and MIX) in comparison to a commercial-like diet (CTRL). A ^1^H-nuclear magnetic resonance (NMR) spectroscopy was used to assess the metabolic profiles of muscle and liver tissue after feeding the fish the experimental diets for 16 weeks. The comparative approach revealed a decrease in metabolites that are associated with energy deficiency in both tissues of fish fed with fishmeal-reduced diets compared to the commercial-like diet (CTRL). Since growth and feeding performance were unaffected, the observed metabolic response suggests that the balanced feed formulations, especially at lower fishmeal replacement levels, have the potential for industry application.

## 1. Introduction

Research on aquafeeds has identified the production of fishmeal (FM), soybean and wheat, which are the main protein sources in current feed formulations that contribute to eutrophication and climate change [1,2]. Therefore, a reduction in the FM content and the integration of sustainable plant and other alternative protein sources would improve the environmental performance of turbot farming. The use of circular-economy (CE) driven feed ingredients based on other protein sources available in Europe, such as rapeseed, pea, microbial meals, and land-animal proteins, can reduce the conflict of use with human nutrition, diversify supply, and provide a viable alternative to FM and soybeans [3]. All of the above-mentioned alternative feed ingredients have been assessed in various feeding trials with high-trophic fish focusing on key performance indicators, such as growth, feed conversion, nutrient retention, digestibility, and somatic indices [4,5,6,7,8,9]. However, turbot is strictly carnivorous during its life cycle [10] and has a high demand for protein (55%) [11] and, depending on the alternative protein sources used, significant effects on growth, feed performance and organismic parameters were observed at replacement levels of more than 30–35% for juvenile turbot [12,13,14,15]. However, effects in organs, tissues, transcriptome, and metabolome can be detected even before the performance indicators are affected [16,17,18,19]. Among the ‘omics’ approaches, metabolomic studies are attracting increasing interest in aquaculture research in order to gain a deeper understanding of how feed ingredients affect performance indicators through changes in the physiological process [20]. Nuclear magnetic resonance (NMR) spectroscopy is a powerful analytical tool used for quantitative metabolic profiling providing information about the overall metabolic state of an organism [21,22,23]. In high-trophic fish species, different metabolic pathways for glucose and amino and fatty acids are affected by full plant-based diets [16] or diets based on alternative ingredients, such as land-based proteins [24,25], single-cell proteins [26], or fish protein hydrolysates [27]. Thereby, the liver and muscle are good target tissues for diet-dependent changes in metabolic profiles [28]. The liver performs a key role in utilizing and storing ingested energy, deposited as glycogen and lipid. On the other hand, muscle tissue is the main location in a fish’s body, where the energy is used for locomotion and growth. However, many metabolomics studies in aquaculture fish did not regard the potential of balanced feed formulations to counteract the negative effects on the metabolome as observed with graded-level single ingredients diets [24,25,27,29].

In a preceding feeding trial [7], growth and feed performance were not affected or only slightly affected, whereas the nutritional status was affected by the feed formulations. To elucidate the effects of the protein sources and the level of FM replacement on the metabolic profile, in this study, a diet with a balanced mixture and higher level of FM replacement was included. Using ^1^H-NMR spectroscopy, this study assessed diet-dependent changes in metabolites of muscle and liver tissue of juvenile turbot (*Scophthalmus maximus*) after the fish were fed with three eco-efficient feed formulations, including plant and animal protein sources, and emerging protein sources and two graded FM replacement levels.

## 2. Materials and Methods

### 2.1. Experimental Setup and Design of Feeding Trial

The feeding experiment was carried out in the Centre for Aquaculture Research (ZAF) at the Alfred Wegener Institute for Polar and Marine research in Bremerhaven, Germany. The juvenile turbot (*Scophthalmus maximus*) were purchased from France Turbot (L’Épine, France) and acclimated to the recirculating aquaculture system (RAS) for 2 weeks prior to starting the 16 weeks experimental trial. The RAS consisted of 36 tanks with a bottom area of one m^2^ and a volume of 700 L, and the water processing consisted of a drum filter, ozone treatment, protein skimmer, and a nitrifying and a denitrifying biofilter. The physical parameters of the process water were monitored constantly (temperature, 16.4 ± 0.2 °C; pH, 7.6 ± 0.1; conductivity, 52.1 ± 1.3 mS cm^−1^; and oxygen saturation, 103.5 ± 4.3%; SC 1000 Multiparameter Universal Controller, Hach Lange GmbH, Düsseldorf, Germany). The concentrations for N-ammonium (0.2 ± 0.1 mg L^−1^), N-nitrite (0.5 ± 0.3 mg L^−1^), and N-nitrate (155.1 ± 37.0 mg L^−1^) were measured with the twice a week (QuAAtro39 AutoAnalyzer, SEAL Analytical, Norderstedt, Germany).

All experimental diets were formulated to be isonitrogenous (530 g kg^−1^) and extruded as 3 mm pellets in a floating mode at SPAROS LDA (Olhão, Portugal). Three eco-efficient feed formulations with graded fishmeal (FM) content were tested against a control diet (CTRL), mimicking a typical current commercial formulation used for turbot. The CTRL diet contained conventional levels of FM (500 g kg^−1^), wheat gluten (110 g kg^−1^), and soy protein concentrate (100 g kg^−1^) as the main protein sources. In the eco-efficient feed formulations, FM and fish protein hydrolysates from fishery by-products (by-catch) was in the remaining FM contingent. The soybean ingredients and the remaining protein fraction was supplemented from emerging ingredient sources, such as insect meal, microbial meal, and pea protein. In two diets, plant protein and microalgae (PLANT) and processed animal protein (PAP), respectively, replaced 20% of the FM. In the third diet, a mixture of processed animal protein, plant protein, cell meals, insect meal, and microalgae (MIX) replaced 40% of FM. Furthermore, in all eco-efficient feed formulations, DHA-rich algae and rapeseed oil replaced 60% of fish oil.

The concepts were based on cost-efficient ingredients, such as processed animal proteins (PAP) or consumer-oriented ingredients containing plant-based pea protein concentrate (PLANT) without PAPs, evading consumers’ food safety concerns related to the use of PAPs. The MIX concept should represent a balanced mixture of the other two concepts. Other ingredients, which are currently emerging in the animal feed production, such as insect meal, microbial meal, and microalgae, have a beneficial nutritional profile. However, these emerging ingredients have high, not yet commercially viable prices.

The content of the respective experimental diets is shown in Table 1.

For the experiment, 1000 turbot were weighed (initial mean body weight of 20.4 ± 0.6 g), measured (initial mean body length 10.1 ± 0.1 cm), and randomly (haphazardly) distributed to 20 tanks (50 individuals per tank, 5 tanks per diet) with the same baseline characteristics. The fish were hand fed twice a day (9 a.m. and 2 p.m.) ad libitum for a period of 16 weeks. The growth and feed performance and nutritional status of the fish from all experimental groups were determined as at the end, final body weight (BW) and total body length (BL) were recorded from all remaining fish to calculate the specific growth rate (SGR) as follows:

SGR = 100 × (ln[final BW] − ln[initial BW])/growth days,(1)

The feed conversion ratio (FCR) was calculated from the total feed intake (FI) per fish during growth period divided by the weight gain as follows:

FCR = FI/(final BW − initial BW),(2)

The effects of the two feed formulations, PLANT and PAP, on growth and feed performance and the nutritional status was reported in a previous publication [9].

### 2.2. Tissue Collection and Sample Preparation

At the end of the experiment, 3 fish per tank (15 fish per diet) were sacrificed for tissue sampling. The fish were anaesthetized with 500 mg L^−1^ tricaine methanesulfonate (MS-222; Sigma Aldrich, Darmstadt, Germany). After recording body weight (precision 0.01 g) and body length (precision 0.5 cm), fish were killed by the separation of the gill artery, and the liver and muscle tissue were rapidly sampled on ice; the liver was weighed (precision 0.001 g). The tissues were shock frozen in liquid nitrogen and stored at −80 °C until further analysis. The hepato-somatic index (HSI) is the liver weight divided by the body weight of the sampled fish. The experimental diets were sampled from freshly opened bags and stored at −80 °C until further analysis.

The sample preparation was adapted and performed according to Lannig et al. [30]. In short, feed, muscle, and liver samples were ground under liquid nitrogen and approx. 200–250 mg tissue was homogenized in 5x volume of ice-cold 0.6 M perchloric acid (PCA) (*w*:*v*). After one cycle of 20 s at 6000 rpm and 3 °C, using Precellys 24 (Bertin Technologies, Montigny-le-Bretonneux, France), samples were sonicated for 2 min at 0 °C and 360 W (Branson Sonifier 450, FisherScientific, Schwerte, Germany). Tissue homogenates were divided for analysis of glycogen content and metabolite profile. Glycogen content was determined following the procedure described by Keppler and Decker [31], photometrically after enzymatic hydrolysis of glycogen to glucose. Detailed steps and calculations and the glycogen content in liver and muscle tissue of the fish from the CTRL, PLANT, and PAP groups are described in Hoerterer et al. [7].

### 2.3. Untargeted ^1^H-NMR Based Metabolic Profiling

Homogenates of the experimental diets, muscle and liver tissues were centrifuged for 2 min at 0 °C and 16,000× *g*, and supernatants were neutralized with ice cold KOH and PCA to pH 7.0–7.5. To remove precipitated potassium, perchlorate samples were centrifuged again for 2 min at 0 °C and 16,000× *g*. The entire supernatant was transferred, shock-frozen in liquid nitrogen, and stored an −80 °C for later analysis. For NMR spectroscopy analyses, samples were defrosted and dried in a rotational vacuum concentrator (RVC 2–18 HCl, Christ GmbH, Osterode am Harz, Germany) at room temperature overnight. Afterwards, samples were re-suspended 1:1 (*w*:*v*) in deuterated water (D_2_O) containing 0.05% of trimethylsilyl propionate (TSP) (45010, Sigma Aldrich, St. Louis, MA, USA) to a final concentration of 1 g/mL of the original frozen sample weight. TSP was used as a chemical shift and quantification standard. The resuspended samples were centrifuged for 10 min at room temperature and 16,000× *g*, for each sample 45 μL of the supernatant were transferred into NMR needle tubes (1.7 mm capillary tube, FisherScientific, Schwerte, Germany). One-dimensional ^1^H-NMR spectra for feed and tissues extracts were acquired using a vertical 9.4 T wide bore magnet with Avance III HD (Bruker-GmbH, Ettlingen, Germany) at 400.13 MHz with a 1.7 mm diameter triple tuned (^1^H-^13^C-^15^N) probe [32]. The samples were measured using a Carr–Purcell–Meiboom–Gill (Bruker protocol cpmgpr1d, TOPSPIN 3.5, Bruker GmbH, Ettlingen, Germany) with water suppression at room temperature using the following parameters: acquisition time (AQ), 4.01 s; sweep width (SW), 8802 Hz (22 ppm); delay (D1), 4 s; dummy scan (DS), 4; and number of scans (ns), 128. Each spectrum was processed and analyzed with Chenomx NMR Suite 8.4 software (Chenomx Inc., Edmonton, Canada). Before analyzing, the spectra were corrected for phase, shim and baseline and calibrated to TSP signal (at 0.0 ppm). Representative spectra for each diet are shown in Supplementary Materials Figures S1–S4. The specific metabolites of the processed spectra were confirmed by structure using the internal database of Chenomx and checked for reliability with the literature data available for aquaculture fish [16,24,33,34] (confidence level 2 [35]). The Chenomx software provided the concentration of the assigned metabolites based on the concentration of the internal standard TSP [36]. In total, signals of 31 compounds were annotated in the ^1^H-NMR spectra of the experimental diets, 29 in liver, and 33 in muscle extracts (Supplementary Materials Table S1).

### 2.4. Statistical Analysis

The fish performance parameters for growth and feed utilization parameters and the condition factor were calculated as means of all fish per tank with five tanks per treatment. The sampled fish per treatment were considered as individual data points for the organ indices, the glycogen and glucose contents, and the metabolite concentrations in the muscle and liver (N = 15 per diet). Metabolite concentrations in the liver and muscle tissue extracts of fish fed the experimental diets (CTRL, PLANT, PAP, and MIX) were analyzed using univariate and multivariate statistical analysis. The metabolite concentrations were transformed by applying a generalized log-transformation to stabilize the variance across the detected metabolite concentrations [37]. Unsupervised principle component analysis (PCA) and Supervised partial least-squares discriminant analysis (PLS-DA) were applied using the Metaboanalyst 5.0 web application [38].

Fish performance parameters, individual nutritional parameters, and the metabolites were analyzed using SigmaPlot (SigmaPlot 12.5, Systat Software Inc., Palo Alto, CA, USA). One-way ANOVA (overall significance level was *p* < 0.05) with post hoc Holm–Sidak method for all pairwise multiple comparison procedures was performed to detect and validate differences between the experimental groups (CTRL, PLANT, PAP, and MIX). Values are given as means ± standard deviation (SD). As the metabolite concentrations in the muscle and liver tissues were not normally distributed, the medians of diets were tested for significance with Kruskal–Wallis analysis (*p* < 0.05) and compared with Tukey. For differences between the different levels of FM replacement, the groups were defined as CTRL = 0%, PLANT and PAP = 20%, respectively, and MIX = 40%.

The experiments were conducted under the guidelines of the local authority, and the animal study protocol was approved by the local authority ‘Food surveillance, animal welfare and veterinary service (LMTVet)’ of the state of Bremen (500–427-103–1/2019–1-19).

## 3. Results

This study evaluated how three different feed formulations (PLANT, PAP, and MIX) affected the metabolic profile of juvenile turbot fed for 16 weeks with 0% mortalities. The diets aimed to be isoenergetic and isolipidic; however, unexpected variations in the raw material composition led to varied crude lipid (+9% in PLANT and −18% in MIX) and gross energy content (+9% on PAP and +12% in MIX) compared to the CTRL diet.

To link the response in the metabolic profile to the growth and feed performance and the nutritional status of the fish, the performance of the MIX group (new data set) is presented in comparison to the performance of CTRL, PLANT, and PAP groups (data set previously published in Hoerterer et al. [7]).

### 3.1. Growth and Feed Performance and Nutritional Status of Fish Fed the Different Diets

The growth and feed performance of the fish from the MIX group were not significantly affected by the diet and did not significantly differ from the fish of other experimental groups (see Supplementary Table S2, [7]). Even though it is not significantly different, after 16 weeks, fish from the CTRL group had overall the best growth and feed conversion followed by the fish from the PLANT and PAP groups, and the fish from the MIX group having the lowest performance.

The sampled fish (n = 15 per dietary treatment) had similar final body weight (see Supplementary Table S3). Hepato-somatic indices (HSI) were significantly lower in fish from the MIX group (1.4 ± 0.2) than in fish from the CTRL group, with no significant differences to the PLANT and PAP groups ([7]; One-Way ANOVA, *p* = 0.006).

The glycogen and glucose levels in the muscle of fish from the MIX group (1.6 ± 0.7 mg/g, 0.12 ± 0.04 mg/g, respectively) were not significantly different to the fish from the CTRL, PAP, and PLANT groups (Supplementary Table S3, [7]). In contrast, the hepatic glycogen content in fish from the MIX group (41.6 ± 22.5 mg/g) was significantly lower than the fish from the CTRL group with no significant differences to the fish from the PAP and PLANT groups ([7]; One-way ANONA, *p* = 0.025). Even though it is not significant, the hepatic free glucose was highest in the fish from the MIX group, leading to a significantly two-fold higher hepatic glucose/glycogen ratio compared to the fish from the CTRL, PAP, and PLANT groups (One-way ANOVA, *p* = 0.009). Additionally, the level of FM replacement had significant effect on the HSI and hepatic glycogen, with significantly higher values in the fish from the CTRL group compared to the fish from the PLANT and PAP (20% FM replacement) and MIX (40% FM replacement) groups (One-Way ANOVA, post hoc Holm–Sidak method, *p* = 0.006, *p* = 0.002, respectively).

### 3.2. Patterns of Compounds in Feed

In the control and the three experimental diets, 31 compounds were detected (see Table 2). Most compound concentrations did not differ between the diets, but NMR spectroscopy nicely highlighted the supplementation of methionine and taurine, showing approx. three- to ten-fold higher concentrations in the PLANT, PAP, and MIX diets compared to the CTRL diet. Even though betaine (in the form of betaine HCl) was supplemented to the diets, the betaine concentrations did not differ much between the diets. Creatine phosphate, lactate, N,N-dimethylglycine, and O-phosphocholine had at least two-fold higher concentrations in the experimental diets than in the CTRL diets. Only sarcosine concentrations were apparently two times higher in the CTRL diets compared to the experimental diets.

### 3.3. ^1^H-NMR Based Metabolic Profile of the Muscle and Liver Tissue

Univariate and multivariate statistical analysis were used to detect differences in the tissue metabolite concentrations between fish fed the different diets and the level of FM replacement (CTRL = 0%, PLANT and PAP = 20%, and MIX = 40%). The composition of assigned metabolites in the muscle tissue did not differ between the experimental groups, except for varying compound concentrations.

**Figure 1:**
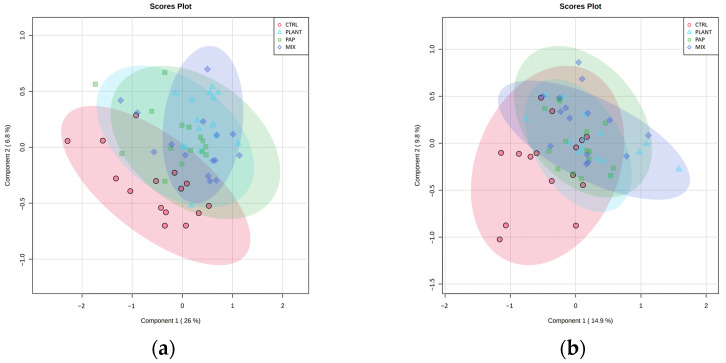
PLS-DA model for the concentrations of assigned metabolites determined in aqueous tissue extracts of juvenile turbot (*Scophthalmus maximus*) fed with 4 experimental diets for 16 weeks (n = 15 fish per diet). (**a**) Score plot of individuals on first 2 components of 33 metabolites in the muscle tissue; and (**b**) Score plot of individuals on first 2 components of 29 metabolites in the liver tissue. Ellipses correspond to a confidence interval of 95% for each group. Red circles, CTRL: commercial-like formulation, Blue triangles, PLANT: plant protein, Green squares, PAP: processed animal protein, purple diamonds, MIX: mixture of processed animal and plant protein.

The supervised partial least squares discriminant analysis (PLS-DA) score plot (Figure 1a) highlights the difference between the metabolic compounds found in the muscle samples. The fish from the CTRL group are distinctive from the fish from the other groups (clearly visible in the 3D-scatterplot), whereas the fish from the MIX group over span the plots of the fish from the PLANT and PAP groups, showing that mixing the alternative feed ingredients could balance deficiencies in the plan-based and animal-based ingredients. The score plots PLS-DA of the liver tissue show no separation between the dietary groups (Figure 1b). The PLS-DA score plot for the FM replacement groups (see Supplementary Material Figure S5) shows separation between the metabolic compounds in the muscle of the fish from the CTRL group and the other replacement groups, placing the 40% farther away.

### 3.4. Diet-Dependent Differences in Metabolic Profile in the Muscle and Liver Tissue

**Figure 2:**
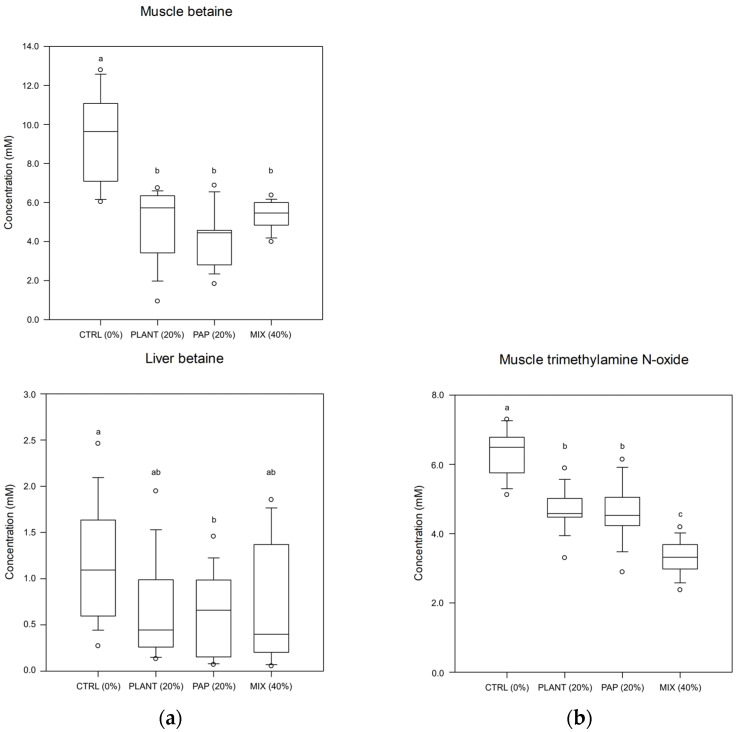
Box plots of metabolite concentrations in aqueous tissue extracts of juvenile turbot (*Scophthalmus maximus*) fed with 4 experimental diets for 16 weeks (n = 15 fish per diet): (**a**) Metabolite concentrations significantly affected by the dietary formulation; (**b**) Metabolite concentrations significantly affected by the level of fishmeal replacement. For each plot, medians of diets were tested for significance with Kruskal–Wallis analysis (*p* < 0.05) and compared with Tukey. Different letters (a, b, c) indicate significant differences between treatment groups. CTRL: commercial-like formulation, PLANT: plant protein, PAP: processed animal protein, MIX: mixture of processed animal and plant protein, 0%, 20%, and 40% level of fishmeal replacement.

The muscle extracts of fish from the CTRL group had a significantly higher concentration of betaine (Kruskal–Wallis analysis; *p* < 0.001) than muscle tissue of fish from the other groups (PLANT, PAP, and MIX; Figure 2a) with no significant differences related to the level of FM replacement (Kruskal–Wallis analysis, *p* > 0.05). In contrast, the concentration of trimethylamine N-oxide (TMAO) in muscle tissue significantly decreased with the increasing level of FM replacement, with fish from the CTRL group having the highest, fish from the PLANT and PAP groups (20% FM replacement) intermediate, and fish from the MIX group the lowest concentrations (Kruskal–Wallis analysis; *p* < 0.001, Figure 2b). In liver tissue, betaine concentration was significantly lower in fish from the PAP group compared to the fish from the CTRL (Kruskal–Wallis analysis; *p* = 0.026, Figure 2a). When analyzed by the FM replacement, the betaine concentrations were significantly highest in fish fed the CTRL (median 1.1 mM) compared to fish fed the MIX diet (40%; median 0.66 mM) and fish fed with the PLANT and PAP (20%; median 0.6 mM) (One-way ANOVA, *p* = 0.010).

## 4. Discussion

In need of alternatives to traditional feed ingredients, such as FM and soybean, feeds with ingredients from the circular economy offer the opportunity for sustainable development of European aquaculture. The present study evaluated the metabolic response in liver and muscle tissue of juvenile turbot to three eco-efficient feed formulations at two levels of FM replacement.

### 4.1. Growth and Feed Performance

The present results suggest that fish-derived ingredients from by-products in combination with plant- and terrestrial animal-based ingredients allow for a complete replacement of traditional feed ingredients and a reduction of 40% FM without compromising growth and feed performance. This is a progress compared to the literature, where decreased growth and feed performance were observed when more than 30–35% of FM was replaced with single ingredients, such as land-based protein sources [12,13,39,40,41,42,43].

### 4.2. Metabolic Response in the Muscle

In the muscle tissue of the juvenile turbot, the formulations and the level of FM replacement negatively affected betaine and trimethylamine N-oxide (TMAO) concentrations. Betaine and TMAO are acting as osmolytes and are linked to the choline and methionine cycle. Osmolytes are often the main metabolite groups detected in the aqueous extracts of tissues from marine fish [44]. In this study, betaine concentrations in the muscle tissue were not correlated to the level of FM replacement but were clearly reduced by more than 40% in the fish fed with the eco-efficient feed formulations compared to the fish from the CTRL group. Betaine is known to be decreased by 50% in the muscle tissue of fasted rainbow trout (*Onchorhynchus mykiss*) [45] and by 25% in red drum (*Sciaenops ocellatus*) fed with soybean-based diets [16]. The supplementation of betaine HCl (5 g kg^−1^) in the three eco-efficient feed formulations to act as a feed attractant seems not to have had any influence on the betaine concentrations in the muscle. Both betaine and choline, precursor of betaine, have similar levels in all experimental diets giving the same baseline to all groups. In contrast to betaine, TMAO is correlated to the level of FM replacement with a 30% decrease at 20% FM replacement (PLANT and PAP groups) and 50% reduction at 40% FM replacement (MIX group) compared to the fish from the CTRL group. Similar to this study, TMAO was decreased by 78% in muscle tissue of red drum fed with soybean-based diets [16]. In contrast, TMAO was increased in the muscle of European Seabass fed with diets containing raw starch [33]. The role of betaine and especially TMAO as a marker for dietary manipulation needs to be further investigated since the response can strongly differ. Melis et al. [46] suggested that TMAO might be a molecular marker for increased metabolic activity in their study due to thermal stress. Furthermore, TMAO level seem to be correlated to the stored lipid content in the body [47], with fish from the MIX group having the lowest crude lipid content (3.2%) compared to the fish from the other groups [7]. This supports the assumption that decreased betaine and TMAO concentrations in the muscle tissue of turbot could indicate energy deficiency caused by the diets resulting in reduced metabolic activity and body lipid content. TMAO levels might be underestimated due to the potential impact of non-enzymatic degradation during storage [48]. However, in this study, the possible degradation can be considered small as all samples were stored in the same way and analysis was conducted in in random order.

### 4.3. Dietary Effects on Energy Storage, Glucose Metabolism, and Metabolic Profile in Liver

In contrast to unaffected growth and feed performance, the diet with a balanced mixture of processed animal protein, plant protein, cell meals, insect meal, and microalgae (MIX) had a negative effect on the hepatic nutritional status of fish seen by decreased HSI and glycogen levels, most likely being correlated to the reduced FM and energy content in the diet. Both HSI and hepatic glycogen content are positively correlated in turbot [49,50,51,52] as hepatic glycogen level serves as an energy storage in most fish species [53]. Compared to marine-based diets, plant-based diets seem to modulate glycolysis and gluconeogenesis in fish liver [28]. In this study, hepatic glucose content was not diet-dependent; however, the higher ratio of glucose to glycogen suggests that the glycogenolysis/gluconeogenesis was affected in the liver of the fish from the MIX group. A higher glucose/glycogen ratio could indicate that glucose was mobilized from stored glycogen as a first response to energy deficiency [28]. Decreased hepatic glycogen, together with increased hepatic glucose content, was observed in turbot when fed with plant-based diets [27]. Energy deficiency could be caused by decreased availability and digestibility of dietary nutrients, such as protein [7], energy, and presumably lipids in the eco-efficient feed formulations. As the experimental diets contained a variable crude lipid content and different ratios of lipid sources, the effects of substrate preference for different lipid classes in oxidation might perform a role in supposed energy deficiency [54] and needs to be further elucidated. This might have led to the mobilization of glucose from glycogen as a source of energy [45,55], resulting in a decreased storage capacity of hepatic glycogen content accompanied by significantly lowered his in turbots fed the MIX diet compared to CTRL fish.

^1^H-NMR-based metabolic profiling revealed significantly reduced betaine concentrations in the liver of juvenile turbot fed with the PAP diet by 60% compared to the fish from the CTRL group. As reviewed by Roques et al. [28], the choline cycle can be affected in fish fed diets with plant-based ingredients leading to altered choline, betaine, N, N-dimethylglycine, dimethylamine, and O-phosphocholine concentrations. The choline cycle is linked to the lipid metabolism, and alterations are an indicator for large differences in the lipid composition [28] and unbalanced supply of other methyl donors, such as methionine [56], as it is reflected by the TMAO levels in the muscle tissue [47]. The decrease in betaine could be linked to the significantly lower whole body lipid of the turbot fed with the PAP diet [7]. However, the difference is small, and the results showed that the alternative formulations with various sources for lipids (CE-salmon oil, rapeseed, and microalgae) and the supplemented methionine (3 g kg^−1^) balanced possible deficiencies in the single ingredients used. Furthermore, there were no effects detected on metabolic compounds related to amino acid catabolism, such as leucine and valine [24,34,57], suggesting that the eco-efficient feed formulations are suitable to balance the amino acid profiles of the single ingredients with the usual amino acids supplemented to the diet. This shows that the concept of using protein sources of varying origin did not affect the amino acid metabolism as is was shown in other studies with single ingredients tested [16].

### 4.4. Metabolites as Markers of Alteration of Metabolism Induced by Eco-Efficient Feed Formulations

Notably, in this study, only the concentrations of glycogen, betaine, and TMAO were significantly affected by the experimental diets, whereas other annotated metabolites, such as creatine, lactate, glutamine, di-methyl-glycine, and valine, showed no diet-dependent alterations. Roques et al. [28] reviewed metabolomic studies investigating the effects of dietary incorporation of plant proteins on aquaculture fish and summarized affected metabolic pathways related to the above-mentioned metabolites. As this study’s experimental diets were designed to meet the species demand for all essential nutrients and since the growth and feed performance was not affected, we expected only small differences in the ^1^H-NMR-based metabolic profile. The level of FM replacement (20% for PLANT and PAP) was low compared to other experimental studies in which significant impairment of the growth performance was observed at 30–35% FM replacement [12,13,14,15,39,58,59]. The level of FM replacement of 40% in the MIX group was above the above-mentioned threshold; however, no negative effects on growth and feed performance were observed in this study. As the fish from the MIX group had a similar nutritional status as the fish from the PLANT and PAP group, despite the high FM replacement, this could indicate that turbot fed with the MIX formulation could have a better performance than the turbot from the PLANT and PAP groups. Furthermore, higher levels of FM replacement might lead to a clearer picture on the effects of the eco-efficient feed formulations on the metabolic profile of turbot as it was shown for inclusion levels of insect meal in rainbow trout [24], soybean meal in red drum [29], or FM replacement in cobia [25].

## 5. Conclusions

This study’s results highlight that ^1^H-NMR-based metabolic profiles are a suitable tool to detect early alterations in the metabolism of juvenile turbot related to decreased FM levels and eco-efficient feed formulations based on alternative protein sources before growth and other physiological parameters are affected. In contrast to preceding research on alternative fish feed ingredients, this study showed that feeding turbot with a balanced mixture of alternative feed ingredients instead of one single ingredient does not alter the metabolic response as strongly as it would be expected at a high level of FM replacement. These effects perform an important role when the feed formulations are applied to commercial aquaculture. With additional environmental stressors, such as changing temperatures, water quality, and pathogens, these effects might result in reduced growth and feed performance, which in turn can lead to stress and its associated negative effects. Especially for turbot, NMR-based metabolomic studies could lead to a better understanding of dietary manipulations, circadian rhythm, and stress response. An early detection of negative effects starting on the metabolic level could enable fish farmers to prevent reduced fish performance. The establishment of non-lethal (plasma) and non-invasive (mucous or feces) methods to assess and monitor the metabolic response of fish to various environmental factors could lead to increased animal welfare and higher production benefits. As this study only focused on the aqueous tissue extracts, lipophilic extract would give a more detailed view on metabolites associated with lipid metabolism. Furthermore, NMR-based metabolomics in plasma are of particular interest as it provided a non-lethal tool to for continuous monitoring in fish, elucidating effects over time. Further investigation on the diet-dependent effects on the metabolome of other life stages of turbot is important. The grow-out phase from 100 g onwards is with approx. 18 months the longest, and alterations in the physiology might have a magnified effect on the growth and feed performance, leading to the economic success of the aquaculture farm. Assessing the effects of eco-efficient feed formulations on the biological level is only one part of improving the environmental performance of aquaculture production. The fish performance can be included in life-cycle assessment for single ingredients or feed formulations.

## Figures and Tables

**Table 1:** Formulation and proximate composition of the experimental diets for juvenile turbot (*Scophthalmus maximus*) on wet weight basis.

	CTRL	PLANT	PAP	MIX
Level of Fishmeal Replacement	0%	20%	20%	40%
Ingredients (g kg^−1^)				
Fishmeal ^1^	500			
Fishmeal (by-product) ^2^		350	350	250
Fish hydrolysate (by-product) ^x^		50	50	50
Insect meal (*Hermetia illucens*) ^x^		50	50	75
Porcine hemoglobin ^3^			25	
Poultry meal ^4^			102	75
Microbial protein meal (*Methanotrophic bacteria*) ^x^		25	25	50
Yeast protein meal (*Saccharomyces cerevisiae*) ^x^		25	25	50
Microalgae meal (*Arthrospira platensis*) ^1^		20		30
Microalgae meal (*Chlorella vulgaris*) ^5^		5		6
Microalgae meal (*Tetraselmis chuii*) ^5^		2		2
Soy protein concentrate ^6^	100			
Pea protein concentrate ^7^		124	50	80
Wheat gluten ^7^	110	115	100	100
Soybean meal ^8^	40			
Wheat meal ^9^	80			
Pea starch ^10^	40	88.9	89.9	89.9
Fish oil ^1^	116	46.4	46.4	46.4
DHA-Rich algae (*Schizochytrium*) ^11^		10.8	10.8	18.8
Rapeseed oil ^12^		46.4	34.4	34.4
Rapeseed lecithin ^13^		8	8	8
Vitamin and mineral premix ^14^	10	10	10	10
Vitamin C ^15^	0.5	0.5	0.5	0.5
Vitamin E ^15^	0.5	0.5	0.5	0.5
Betaine HCl ^16^		5	5	5
Macroalgae mix ^17^		5	5	5
Antioxidant ^18^	1.8	1.8	1.8	1.8
Sodium propionate ^19^	1	1	1	1
L-Tryptophan ^20^		1.5	1.5	1.5
DL-Methionine ^21^		3	3	3
L-Taurine ^16^		5	5	6
Yttrium oxide ^22^	0.2	0.2	0.2	0.2
Proximate composition				
Moisture (%)	4.1	6.7	7.3	7.5
Crude Protein (%)	52.9	52.8	52.8	52.6
Crude Lipid (%)	16.5	18.1	16.2	13.9
Ash (%)	7.1	9.9	10.5	9.4
Energy (MJ kg^−1^)	23.1	21.2	20.8	20.9

CTRL: commercial-like formulation, PLANT: plant protein, PAP: processed animal protein, MIX: mixture of processed animal and plant protein. ^x^ not disclosed; ^1^ Sopropêche, Wimille, France; ^2^ Conserveros Reunidos S.A., A Coruña, Spain; ^3^ SONAC BV, AA Son, The Netherlands; ^4^ SAVINOR UTS, Covelas, Portugal; ^5^ Allmicroalgae, Pataias, Portugal; ^6^ ADM, Amsterdam, The Netherlands; ^7^ Roquette Frères, Lestrem, France; ^8^ CARGILL, Sant Cugat del Vallés, Spain; ^9^ Casa Lanchinha, Alhos Vedros, Portugal; ^10^ COSUCRA, Pecq, Belgium; ^11^ Alltech, Sarney, Dunboyne, Co. Meath, Ireland; ^12^ Henry Lamotte Oils GmbH, Bremen, Germany; ^13^ Novastell, Vernon, France; ^14^ DL-alpha tocopherol acetate, 255 mg; sodium menadione bisulphate, 10 mg; retinyl acetate, 26,000 IU; DL-cholecalciferol, 2500 IU; thiamine, 2 mg; riboflavin, 9 mg; pyridoxine, 5 mg; cyanocobalamin, 0.5 mg; nicotinic acid, 25 mg; folic acid, 4 mg; L-ascorbic acid monophosphate, 80 mg; inositol, 17.5 mg; biotin, 0.2 mg; calcium pantothenate, 60 mg; choline chloride, 1960 mg. Minerals (g or mg∙kg^−1^ diet): copper sulphate, 8.25 mg; ferric sulphate, 68 mg; potassium iodide, 0.7 mg; manganese oxide, 35 mg; organic selenium, 0.01 mg; zinc sulphate, 123 mg; calcium carbonate, 1.5 g; excipient wheat middlings; ^15^ DSM Nutritional Products, DSM Nutritional Products, Switzerland, Switzerland; ^16^ ORFFA, ZL Breda, The Netherlands; ^17^ Ocean Harvest, Gortnaloura, Tuam, Co. Galway, Ireland; ^18^ Kemin Europe NV, Herentals, Belgium; ^19^ Disproquímica, Vialonga, Portugal; ^20^ Ajinomoto EUROLYSINE S.A.S, Paris France; ^21^ EVONIK Nutrition and Care GmbH, Essen, Germany; ^22^ Sigma Aldrich, St. Louis, MA, USA.

**Table 2:** Concentrations (mM) of compounds found in the aqueous extracts from the different diets.

	CTRL	PLANT	PAP	MIX
Level of Fishmeal Replacement	0%	20%	20%	40%
Acetate	23.9	27.9	24.4	22.0
Alanine	7.2	6.9	7.3	8.2
Betaine ^suppl.^	20.1	20.4	20.9	24.2
Carnitine	1.3	1.3	1.3	2.3
Choline	10.5	11.8	11.2	11.1
Creatine	6.7	5.1	5.3	5.0
Creatine phosphate	1.2	3.8	5.7	2.7
Creatinine	8.7	8.0	8.1	7.4
Dimethylamine	10.0	18.1	17.1	11.4
Fumarate	0.1	0.1	0.0	0.1
Glucose-6-phosphate	4.4	4.6	3.9	3.3
Glutamate	6.6	8.3	7.0	9.2
Glycine	5.4	5.4	5.2	5.4
Isoleucine	1.3	3.3	1.3	1.8
Lactate	15.6	23.4	24.7	24.6
Leucine	6.5	6.6	7.1	6.8
Malonate	1.9	2.4	1.8	2.1
Methionine ^suppl.^	1.4	15.1	12.7	14.9
N,N-Dimethylglycine	1.2	3.2	3.2	1.8
O-Phosphocholine	1.2	2.8	3.1	2.5
Sarcosine	4.1	2.1	2.0	1.7
Succinate	2.1	2.2	2.0	2.6
Taurine ^suppl.^	11.8	38.4	35.1	30.5
Threonine	1.9	2.0	1.8	2.1
Valine	2.6	2.3	2.5	3.0

CTRL: commercial-like formulation, PAP: processed animal protein, PLANT: plant protein, MIX: mixture of processed animal and plant protein. Values are shown as means (n = 2); ^suppl.^ compounds were supplemented to the PLANT, PAP and MIX diets.

## Data Availability

The data presented in this study are openly available in PANGAEA (publication in process).

## Supplementary Materials

The following supporting information can be downloaded at: https://www.mdpi.com/article/10.3390/metabo13050612/s1, Table S1: Assigned compounds in the ^1^H-NMR-spectrum muscle (M) and liver (L) tissue of juvenile turbot (*Scophthalmus maximus*) fed with the different diets (F). Table S2. Performance parameters of the juvenile turbot (Scophthalmus maximus) fed with different diets for 16 weeks. Table S3. Final body weight, organ indices, and glycogen and glucose levels in wet tissue of muscle and liver of juvenile turbot (*Scophthalmus maximus*) fed with different diets for 16 weeks. Figure S1: Representative ^1^H-NMR spectrum of muscle tissue from turbot fed with the commercial-like CTRL diet. black: spectrum line, red: Sum line of compounds. Spectrum was generated using CHENOMX v8.4. Figure S2: Representative ^1^H-NMR spectrum of muscle tissue from turbot fed with the commercial-like PLANT diet. black: spectrum line, red: Sum line of compounds. Spectrum was generated using CHENOMX v8.4. Figure S3: Representative ^1^H-NMR spectrum of muscle tissue from turbot fed with the commercial-like PAP diet. black: spectrum line, red: Sum line of compounds. Spectrum was generated using CHENOMX v8.4. Figure S4: Representative ^1^H-NMR spectrum of muscle tissue from turbot fed with the commercial-like MIX diet. black: spectrum line, red: Sum line of compounds. Spectrum was generated using CHENOMX v8.4. Figure S5: Score plot of the PLS-DA model for the concentrations of assigned metabolites from the aqueous tissue extracts of muscle of juvenile turbot (*Scophthalmus maximus*) fed with experimental diets with different fishmeal replacement levels (0%, 20% and 40%). Ellipses correspond to a confidence interval of 95% for each group.

## References

[1] Aubin J., Papatryphon E., Van der Werf H.M.G., Petit J., Morvan Y.M. (2006). Characterisation of the environmental impact of a turbot (*Scophthalmus maximus*) re-circulating production system using Life Cycle Assessment. Aquaculture.

[2] Iribarren D., Moreira M., Feijoo G. (2012). Life Cycle Assessment of aquaculture feed and application to the turbot sector. Int. J. Environ. Res..

[3] Hua K., Cobcroft J.M., Cole A., Condon K., Jerry D.R., Mangott A., Praeger C., Vucko M.J., Zeng C., Zenger K. (2019). The future of aquatic protein: Implications for protein sources in aquaculture diets. One Earth.

[4] Kaiser F., Harloff H.J., Tressel R.P., Kock T., Schulz C. (2021). Effects of highly purified rapeseed protein isolate as fishmeal alternative on nutrient digestibility and growth performance in diets fed to rainbow trout (*Oncorhynchus mykiss*). Aquac. Nutr..

[5] Karapanagiotidis I.T., Psofakis P., Mente E., Malandrakis E., Golomazou E. (2019). Effect of fishmeal replacement by poultry by-product meal on growth performance, proximate composition, digestive enzyme activity, haematological parameters and gene expression of gilthead seabream (*Sparus aurata*). Aquac. Nutr..

[6] Fronte B., Abramo F., Brambilla F., De Zoysa M., Miragliotta V. (2019). Effect of hydrolysed fish protein and autolysed yeast as alternative nitrogen sources on gilthead sea bream (*Sparus aurata*) growth performances and gut morphology. Ital. J. Anim. Sci..

[7] Hoerterer C., Petereit J., Lannig G., Johansen J., Pereira G.V., Conceição L.E.C., Pastres R., Buck B.H. (2022). Sustainable fish feeds: Potential of emerging protein sources in diets for juvenile turbot (*Scophthalmus maximus*) in RAS. Aquac. Int..

[8] Petereit J., Hoerterer C., Bischoff-Lang A.A., Conceição L.E.C., Pereira G., Johansen J., Pastres R., Buck B.H. (2022). Adult European Seabass (*Dicentrarchus labrax*) perform well on alternative circular-economy-driven feed formulations. Sustainability.

[9] Hoerterer C., Petereit J., Lannig G., Johansen J., Conceição L.E.C., Buck B.H. (2022). Effects of dietary plant and animal protein sources and replacement levels on growth and feed performance and nutritional status of market-sized turbot (*Scophthalmus maximus*) in RAS. Front. Mar. Sci..

[10] FAO *Scophthalmus maximus*. Fisheries and Aquaculture Division. https://www.fao.org/fishery/en/culturedspecies/psetta_maxima/en.

[11] Oliva-Teles A., Enes P., Couto A., Peres H., Davis D.A. (2022). 8-Replacing fish meal and fish oil in industrial fish feeds. Feed and Feeding Practices in Aquaculture.

[12] Kroeckel S., Harjes A.G.E., Roth I., Katz H., Wuertz S., Susenbeth A., Schulz C. (2012). When a turbot catches a fly: Evaluation of a pre-pupae meal of the Black Soldier Fly (*Hermetia illucens*) as fish meal substitute—Growth performance and chitin degradation in juvenile turbot (*Psetta maxima*). Aquaculture.

[13] Bonaldo A., Di Marco P., Petochi T., Marino G., Parma L., Fontanillas R., Koppe W., Mongile F., Finoia M.G., Gatta P.P. (2015). Feeding turbot juveniles *Psetta maxima* L. with increasing dietary plant protein levels affects growth performance and fish welfare. Aquac. Nutr..

[14] Chen Z.C., Liu Y., Li Y.X., Yang P., Hu H.B., Yu G.J., Ai Q.H., Xu W., Zhang W.B., Zhang Y.G. (2018). Dietary arginine supplementation mitigates the soybean meal induced enteropathy in juvenile turbot, *Scophthalmus maximus* L. Aquac. Res..

[15] Bai N., Gu M., Liu M., Jia Q., Pan S., Zhang Z. (2019). Corn gluten meal induces enteritis and decreases intestinal immunity and antioxidant capacity in turbot (*Scophthalmus maximus*) at high supplementation levels. PLoS ONE.

[16] Casu F., Watson A.M., Yost J., Leffler J.W., Gaylord T.G., Barrows F.T., Sandifer P.A., Denson M.R., Bearden D.W. (2017). Metabolomics analysis of effects of commercial soy-based protein products in red drum (*Sciaenops ocellatus*). J. Proteome Res..

[17] Batista S., Medina A., Pires M.A., Moriñigo M.A., Sansuwan K., Fernandes J.M.O., Valente L.M.P., Ozório R.O.A. (2016). Innate immune response, intestinal morphology and microbiota changes in Senegalese sole fed plant protein diets with probiotics or autolysed yeast. Appl. Microbiol. Biotechnol..

[18] Øverland M., Sørensen M., Storebakken T., Penn M., Krogdahl Å., Skrede A. (2009). Pea protein concentrate substituting fish meal or soybean meal in diets for Atlantic salmon (*Salmo salar*)—Effect on growth performance, nutrient digestibility, carcass composition, gut health, and physical feed quality. Aquaculture.

[19] Glencross B., Evans D., Hawkins W., Jones B. (2004). Evaluation of dietary inclusion of yellow lupin (*Lupinus luteus*) kernel meal on the growth, feed utilisation and tissue histology of rainbow trout (*Oncorhynchus mykiss*). Aquaculture.

[20] Alfaro A.C., Young T. (2018). Showcasing metabolomic applications in aquaculture: A review. Rev. Aquac..

[21] Samuelsson L.M., Förlin L., Karlsson G., Adolfsson-Erici M., Larsson D.G. (2006). Using NMR metabolomics to identify responses of an environmental estrogen in blood plasma of fish. Aquat. Toxicol..

[22] Cappello T., Giannetto A., Parrino V., Maisano M., Mauceri A., Fasulo S. (2019). NMR-based metabolomics: A holistic approach for monitoring complex biological systems. Atti Della Accad. Peloritana Dei Pericolanti-Cl. Sci. Med. Biol..

[23] Kaneko G., Ushio H., Ji H. (2019). Application of magnetic resonance technologies in aquatic biology and seafood science. Fish. Sci..

[24] Roques S., Deborde C., Guimas L., Marchand Y., Richard N., Jacob D., Skiba-Cassy S., Moing A., Fauconneau B. (2020). Integrative metabolomics for assessing the effect of insect (*Hermetia illucens*) protein extract on rainbow trout metabolism. Metabolites.

[25] Schock T.B., Newton S., Brenkert K., Leffler J., Bearden D.W. (2012). An NMR-based metabolomic assessment of cultured cobia health in response to dietary manipulation. Food Chem..

[26] Abro R., Moazzami A.A., Lindberg J.E., Lundh T. (2014). Metabolic insights in Arctic charr (*Salvelinus alpinus*) fed with zygomycetes and fish meal diets as assessed in liver using nuclear magnetic resonance (NMR) spectroscopy. Int. Aquat. Res..

[27] Wei Y., Liang M., Mai K., Zheng K., Xu H. (2017). ^1^H NMR-based metabolomics studies on the effect of size-fractionated fish protein hydrolysate, fish meal and plant protein in diet for juvenile turbot (*Scophthalmus maximus* L.). Aquac. Nutr..

[28] Roques S., Deborde C., Richard N., Skiba-Cassy S., Moing A., Fauconneau B. (2020). Metabolomics and fish nutrition: A review in the context of sustainable feed development. Rev. Aquac..

[29] Casu F., Watson A.M., Yost J., Leffler J.W., Gaylord T.G., Barrows F.T., Sandifer P.A., Denson M.R., Bearden D.W. (2019). Investigation of graded-level soybean meal diets in red drum (*Sciaenops ocellatus*) using NMR-based metabolomics analysis. Comp. Biochem. Physiol. D-Genom. Proteom..

[30] Lannig G., Eilers S., Pörtner H.O., Sokolova I.M., Bock C. (2010). Impact of ocean acidification on energy metabolism of oyster, *Crassostrea gigas*-Changes in metabolic pathways and thermal response. Mar. Drugs.

[31] Keppler D., Decker K., Bergmeyer H.U. (1988). 1.2 Glycogen. Methods of Enzymatic Analysis: Metabolites 1: Carbohydrates.

[32] Georgoulis I., Bock C., Lannig G., Pörtner H.O., Feidantsis K., Giantsis I.A., Sokolova I.M., Michaelidis B. (2022). Metabolic remodeling caused by heat-hardening in the Mediterranean mussel *Mytilus galloprovincialis*. J. Exp. Biol..

[33] Jarak I., Tavares L., Palma M., Rito J., Carvalho R.A., Viegas I. (2018). Response to dietary carbohydrates in European seabass (*Dicentrarchus labrax*) muscle tissue as revealed by NMR-based metabolomics. Metabolomics.

[34] Wei Y., Liang M., Mai K., Zheng K., Xu H. (2017). The effect of ultrafiltered fish protein hydrolysate levels on the liver and muscle metabolic profile of juvenile turbot (*Scophthalmus maximus* L.) by ^1^H NMR-based metabolomics studies. Aquac. Res..

[35] Sumner L.W., Amberg A., Barrett D., Beale M.H., Beger R., Daykin C.A., Fan T.W., Fiehn O., Goodacre R., Griffin J.L. (2007). Proposed minimum reporting standards for chemical analysis Chemical Analysis Working Group (CAWG) Metabolomics Standards Initiative (MSI). Metabolomics.

[36] Schmidt M., Windisch H.S., Ludwichowski K.U., Seegert S.L.L., Pörtner H.O., Storch D., Bock C. (2017). Differences in neurochemical profiles of two gadid species under ocean warming and acidification. Front. Zool..

[37] Purohit P.V., Rocke D.M., Viant M.R., Woodruff D.L. (2004). Discrimination models using variance-stabilizing transformation of metabolomic NMR data. OMICS A J. Integr. Biol..

[38] Chong J., Wishart D.S., Xia J.G. (2019). Using MetaboAnalyst 4.0 for comprehensive and integrative metabolomics data analysis. Curr. Protoc. Bioinform..

[39] Hermann B.T., Reusch T.B.H., Hanel R. (2016). Effects of dietary purified rapeseed protein concentrate on hepatic gene expression in juvenile turbot (*Psetta maxima*). Aquac. Nutr..

[40] Bian F., Zhou H., He G., Wang C., Peng H., Pu X., Jiang H., Wang X., Mai K. (2017). Effects of replacing fishmeal with different cottonseed meals on growth, feed utilization, haematological indexes, intestinal and liver morphology of juvenile turbot (*Scophthalmus maximus* L.). Aquac. Nutr..

[41] Dong C., He G., Mai K.S., Zhou H.H., Xu W. (2016). Palatability of water-soluble extracts of protein sources and replacement of fishmeal by a selected mixture of protein sources for juvenile turbot (*Scophthalmus maximus*). J. Ocean Univ. China.

[42] Fuchs V.I., Schmidt J., Slater M.J., Zentek J., Buck B.H., Steinhagen D. (2015). The effect of supplementation with polysaccharides, nucleotides, acidifiers and *Bacillus* strains in fish meal and soy bean based diets on growth performance in juvenile turbot (*Scophthalmus maximus*). Aquaculture.

[43] Burel C., Boujard T., Kaushik S.J., Boeuf G., Van der Geyten S., Mol K.A., Kuhn E.R., Quinsac A., Krouti M., Ribaillier D. (2000). Potential of plant-protein sources as fish meal substitutes in diets for turbot (*Psetta maxima*): Growth, nutrient utilisation and thyroid status. Aquaculture.

[44] Rebelein A., Pörtner H.O., Bock C. (2018). Untargeted metabolic profiling reveals distinct patterns of thermal sensitivity in two related notothenioids. Comp. Biochem. Physiol. A Mol. Integr. Physiol..

[45] Kullgren A., Samuelsson L.M., Larsson D.G., Björnsson B.T., Bergman E.J. (2010). A metabolomics approach to elucidate effects of food deprivation in juvenile rainbow trout (*Oncorhynchus mykiss*). Am. J. Physiol. Regul. Integr. Comp. Physiol..

[46] Melis R., Sanna R., Braca A., Bonaglini E., Cappuccinelli R., Slawski H., Roggio T., Uzzau S., Anedda R. (2017). Molecular details on gilthead sea bream (*Sparus aurata*) sensitivity to low water temperatures from ^1^H NMR metabolomics. Comp. Biochem. Physiol. a-Mol. Integr. Physiol..

[47] Seibel B.A., Walsh P.J. (2002). Trimethylamine oxide accumulation in marine animals: Relationship to acylglycerol storage. J. Exp. Biol..

[48] Sotelo C.G., Rehbein H. (2000). TMAO-degrading enzymes. Seafood Enzymes.

[49] Miao S.Y., Nie Q., Miao H.J., Zhang W.B., Mai K.S. (2016). Effects of dietary carbohydrate-to-lipid ratio on the growth performance and feed utilization of juvenile turbot (*Scophthalmus maximus*). J. Ocean Univ. China.

[50] Guerreiro I., Enes P., Merrifield D., Davies S., Oliva-Teles A. (2015). Effects of short-chain fructooligosaccharides on growth performance and hepatic intermediary metabolism in turbot (*Scophthalmus maximus*) reared at winter and summer temperatures. Aquac. Nutr..

[51] Zeng L., Lei J.L., Ai C.X., Hong W.S., Liu B. (2015). Protein-sparing effect of carbohydrate in diets for juvenile turbot *Scophthalmus maximus* reared at different salinities. Chin. J. Oceanol. Limnol..

[52] Liu X., Mai K., Liufu Z., Ai Q. (2014). Effects of dietary protein and lipid levels on growth, nutrient utilization, and the whole-body composition of turbot, *Scophthalmus maximus*, Linnaeus 1758, at Different Growth Stages. J. World Aquac. Soc..

[53] Hemre G.I., Mommsen T.P., Krogdahl A. (2002). Carbohydrates in fish nutrition: Effects on growth, glucose metabolism and hepatic enzymes. Aquac. Nutr..

[54] Turchini G.M., Francis D.S., Du Z.-Y., Olsen R.E., Ringø E., Tocher D.R., Hardy R.W., Kaushik S.J. (2022). Chapter 5-The lipids. Fish Nutrition.

[55] Sheridan M.A., Mommsen T.P. (1991). Effects of nutritional state on in vivo lipid and carbohydrate metabolism of coho salmon, *Oncorhynchus kisutch*. Gen. Comp. Endocrinol..

[56] Maruhenda Egea F.C., Toledo-Guedes K., Sanchez-Jerez P., Ibanco-Cañete R., Uglem I., Saether B.S. (2015). A metabolomic approach to detect effects of salmon farming on wild saithe (*Pollachius virens*) populations. J. Agric. Food Chem..

[57] Wagner L., Trattner S., Pickova J., Gómez-Requeni P., Moazzami A.A. (2014). ^1^H NMR-based metabolomics studies on the effect of sesamin in Atlantic salmon (*Salmo salar*). Food Chem..

[58] Bai N., Gu M., Xu X., Xu B., Krogdahl Å. (2017). Protective effects of mannan oligosaccharides on turbot *Scophthalmus maximus* suffering from soy enteropathy. Aquaculture.

[59] Bonaldo A., Parma L., Mandrioli L., Sirri R., Fontanillas R., Badiani A., Gatta P.P. (2011). Increasing dietary plant proteins affects growth performance and ammonia excretion but not digestibility and gut histology in turbot *(Psetta maxima*) juveniles. Aquaculture.

