# Supplementary material for: 1H-NMR-Based Metabolic Profiling in Muscle and Liver Tissue of Juvenile Turbot (Scophthalmus maximus) Fed with Plant and Animal Protein Sources
Source: Metabolites. 2023 Apr 28;13(5):612. doi: 10.3390/metabo13050612 (PMC10221397; doi:10.3390/metabo13050612)
Supplement: Supplementary file 1 [file metabolites-13-00612-s001.zip › Supplementary Materials.pdf]

Supplementary Materials

Table S1: Assigned compounds in the 1H-NMR-spectrum muscle (M) and liver (L) tissue of juvenile turbot (*Scophthalmus maximus*) fed with the different diets (F).

Class	Compound	1H chemical shift (ppm)	Extract
Organic acids	Acetate	1.9	F, M, L
	Adenine	8.2	M
	ADP	8.5	M
Amino acids, dipeptides	Alanine	1.5	F, M, L
	AMP	8.6	M
Amino acids, dipeptides	Anserine	6.8; 3.8	M
Amino acids, dipeptides	Arginine		M
Amino acids, dipeptides	Aspartate	2.8	M
	ATP	8.2; 4.4	M, L
Amines and N-containing compounds	Betaine	3.9; 3.3	F, M, L
Amino acids, dipeptides	Carnitine	3.2	F, M, L
Amines and N-containing compounds	Choline	3.2	F, M, L
Amines and N-containing compounds	Creatine	3.9; 3.0	F, M, L
Amines and N-containing compounds	Creatine phosphate	3.9; 3.0	F, M, L
Amines and N-containing compounds	Creatinine		F, L
Amines and N-containing compounds	Dimethylamine		F, L
	Dimethyl sulfone		F, L
Organic acids	Formate	8.4	M, L
Organic acids	Fumarate	6.5	F, M, L
Sugars	Glucose-6-phosphate	5.2	F, M, L
Amino acids, dipeptides	Glutamate	2.3	F, M, L
Amino acids, dipeptides	Glutamine		F, M
Amino acids, dipeptides	Glycine	3.5	F, M, L
Amino acids, dipeptides	Isoleucine		F, M, L
Organic acids	Lactate	4.1; 1.3	F, M, L
Amino acids, dipeptides	Leucine	0.9	F, M, L
Organic acids	Malonate	3.1	F, M, L
Amino acids, dipeptides	Methionine	2.1	F, M, L
Amines and N-containing compounds	N,N-Dimethylglycine	2.9	F, M, L
Amines and N-containing compounds	O-Phosphocholine		F, L
Amino acids, dipeptides	Ornithine		F
Amino acids, dipeptides	Proline	2.0	F, M
Amino acids, dipeptides	Sarcosine	3.6; 2.7	F, M, L
Amino acids, dipeptides	Serine		F
Organic acids	Succinate	2.4	F, M, L

Amines and N-containing compounds	Taurine	3.4; 3.2	F, M, L
Amino acids, dipeptides	Threonine	1.3	F, M, L
Amines and N-containing compounds	Trimethylamine N-oxide	3.3	F, M
Amino acids, dipeptides	Valine	1.0	F, M, L

Table S2. Performance parameters of the juvenile turbot (Scophthalmus maximus) fed with different diets for 16 weeks.

	CTRL*	PLANT*	PAP*	MIX	P-value
Level of fishmeal replacement	0%	20%	20%	40%	
Initial body weight (g)	20.2 ± 0.3	20.4 ± 0.4	20.1 ± 0.5	20.3 ± 0.4	0.852
Final body weight (g)	85.2 ± 9.7	82.1 ± 9.5	82.9 ± 6.1	81.9 ± 7.3	0.914
Specific Growth rate (SGR)	1.28 ± 0.09	1.24 ± 0.09	1.26 ± 0.06	1.25 ± 0.07	0.852
Feed conversion ratio (FCR)	0.87 ± 0.03	0.90 ± 0.02	0.92 ± 0.03	0.90 ± 0.04	0.109

CTRL: commercial-like formulation, PLANT: plant protein, PAP: processed animal protein, MIX: mixture of processed animal and plant protein. *Underlined data from the CTRL, PAP and PLANT groups were previously published in Hoerterer, et al. [7]; Values are shown as means ± SD (n = 5 tanks per diet), no significant differences were detected by One-way ANOVA ($P > 0.05$).

Table S3. Final body weight, organ indices and glycogen and glucose levels in wet tissue of muscle and liver of juvenile turbot (*Scophthalmus maximus*) fed with different diets for 16 weeks.

	CTRL*	PLANT*	PAP*	MIX	P-value
Level of fishmeal replacement	0%	20%	20%	40%	
Final body weight (g)	89.7 ± 27.2	91.5 ± 33.3	92.1 ± 28.8	87.9 ± 34.3	0.983
Hepato-somatic index (HSI)	1.8 ± 0.3a	1.5 ± 0.3b	1.5 ± 0.3b	1.4 ± 0.2b	0.006
Muscle glycogen (mg g^{-1})	1.7 ± 0.7	2.1 ± 0.7	1.8 ± 0.7	1.6 ± 0.7	0.298
Muscle glucose (mg g^{-1})	0.14 ± 0.05	0.12 ± 0.04	0.14 ± 0.04	0.12 ± 0.04	0.244
Muscle glucose/glycogen	0.12 ± 0.10	0.06 ± 0.03	0.09 ± 0.04	0.08 ± 0.04	0.114
Liver glycogen (mg g^{-1})	63.7 ± 23.9a	46.4 ± 16.0ab	48.0 ± 17.2ab	41.6 ± 22.5b	0.025
Liver glucose (µg g^{-1})	2.9 ± 1.1	2.8 ± 0.6	2.6 ± 0.6	3.2 ± 1.1	0.284
Liver glucose/glycogen ($x10^{-5}$)	5.7 ± 4.7b	6.5 ± 1.9b	6.0 ± 2.1b	11.1 ± 7.8a	0.009

CTRL: commercial-like formulation, PLANT: plant protein, PAP: processed animal protein MIX: mixture of processed animal and plant protein. *Underlined data from the CTRL, PAP and PLANT groups were previously published in Hoerterer, et al. [7]; Values are shown as means ± SD (n = 15 fish per diet), different letters (a, b, c) indicate significant differences between treatment groups detected one-way ANOVA and by Holm-Sidak method ($P < 0.050$).

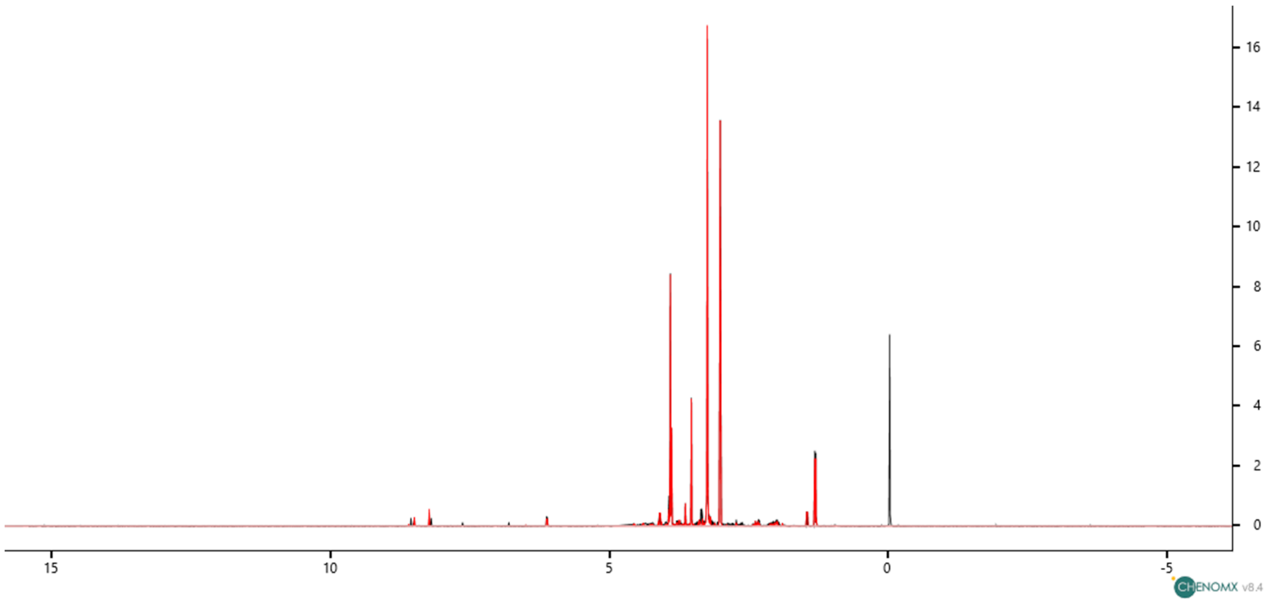

Figure S1: Representative ^{1}H-NMR spectrum of muscle tissue from turbot fed with the commercial-like CTRL diet. black: spectrum line, red: Sum line of compounds. Spectrum was generated using CHENOMX v8.4.

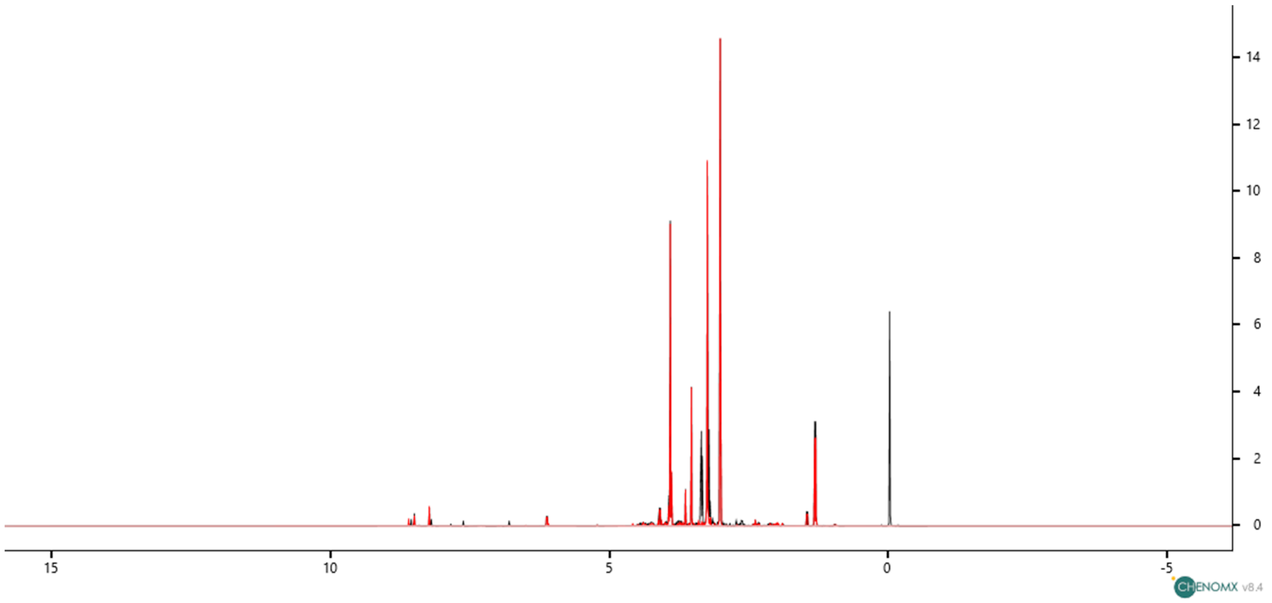

Figure S2: Representative ^{1}H-NMR spectrum of muscle tissue from turbot fed with the commercial-like PLANT diet. black: spectrum line, red: Sum line of compounds. Spectrum was generated using CHENOMX v8.4.

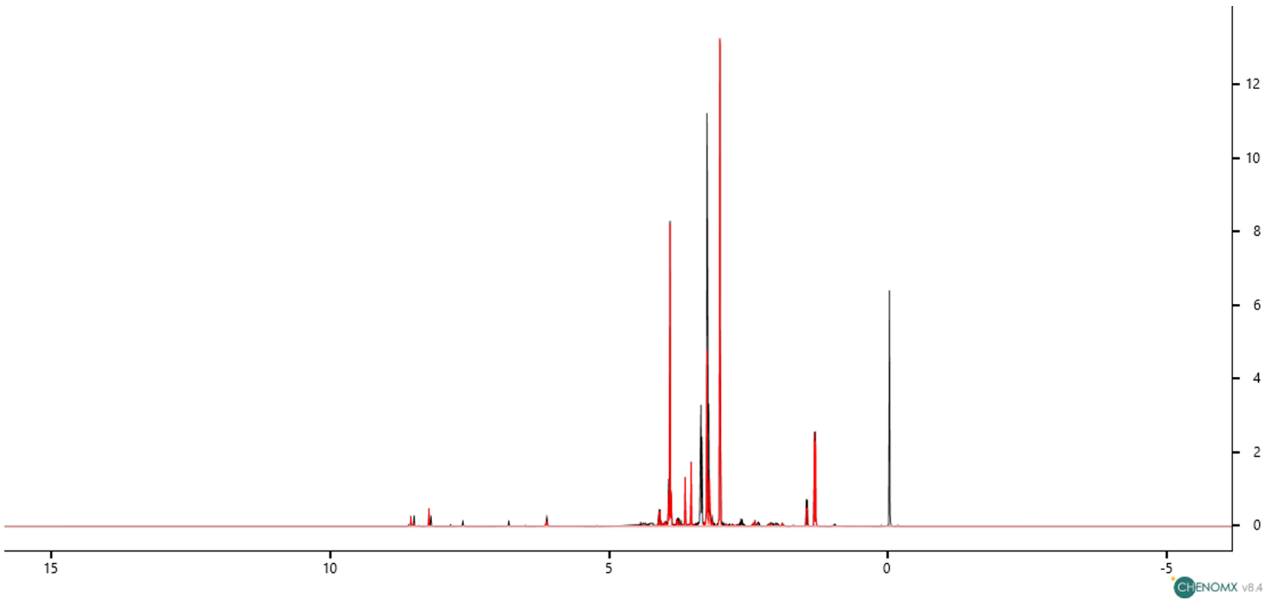

Figure S3: Representative ^{1}H-NMR spectrum of muscle tissue from turbot fed with the commercial-like PAP diet. black: spectrum line, red: Sum line of compounds. Spectrum was generated using CHENOMX v8.4.

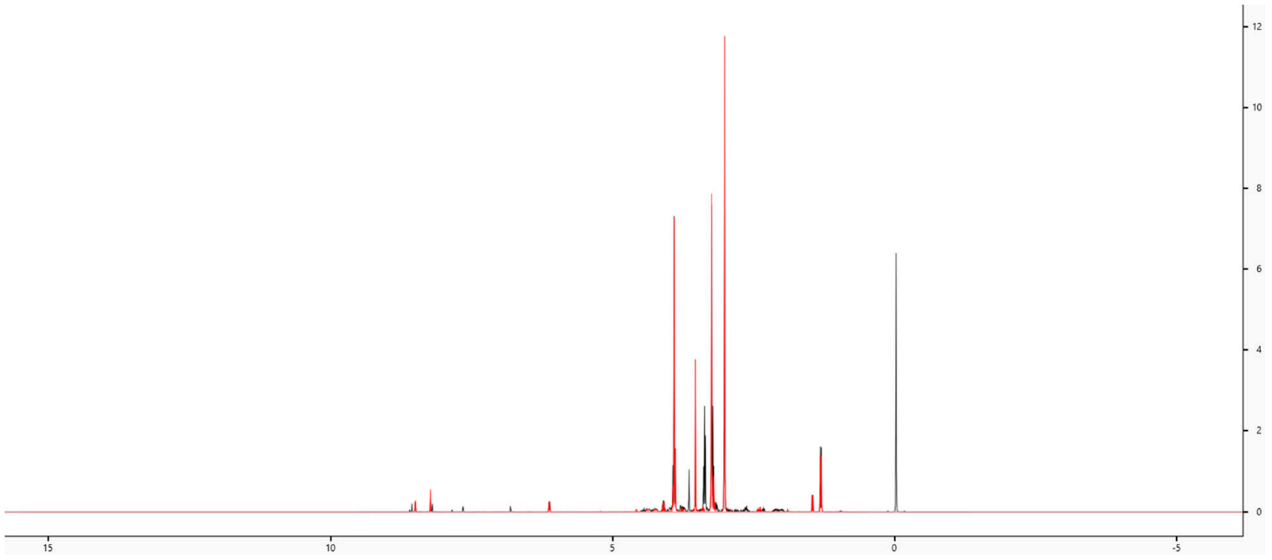

Figure S4: Representative ^{1}H-NMR spectrum of muscle tissue from turbot fed with the commercial-like MIX diet. black: spectrum line, red: Sum line of compounds. Spectrum was generated using CHENOMX v8.4.

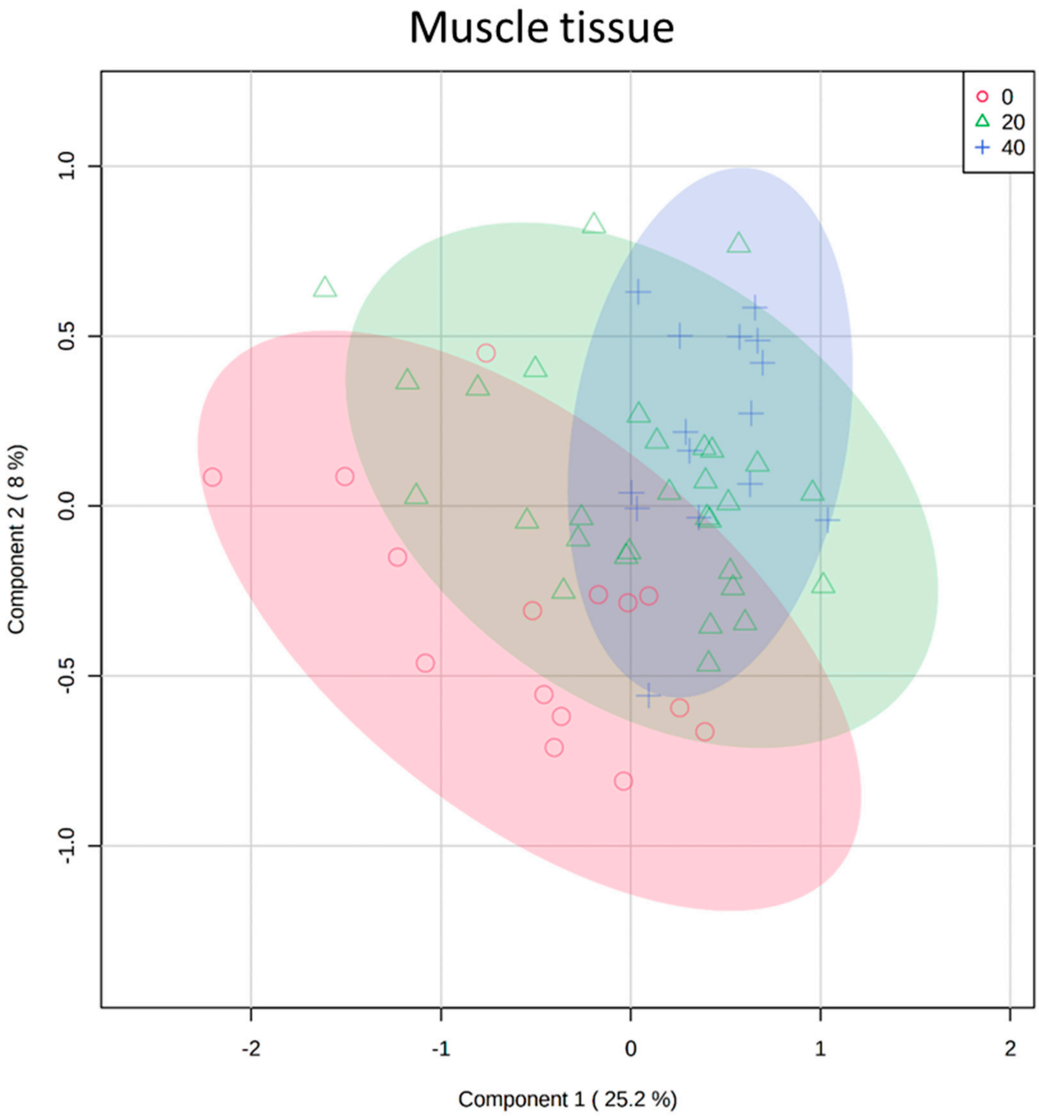

Figure S5: Score plot of the PLS-DA model for the concentrations of assigned metabolites from the aqueous tissue extracts of muscle of juvenile turbot (Scophthalmus maximus) fed with experimental diets with different fishmeal replacement levels (0%, 20% and 40%). Ellipses correspond to a confidence interval of 95% for each group.
