# Supplementary figures and images for: 1H-NMR-Based Metabolic Profiling in Muscle and Liver Tissue of Juvenile Turbot (Scophthalmus maximus) Fed with Plant and Animal Protein Sources
Source: Metabolites. 2023 Apr 28;13(5):612. doi: 10.3390/metabo13050612 (PMC10221397; doi:10.3390/metabo13050612)

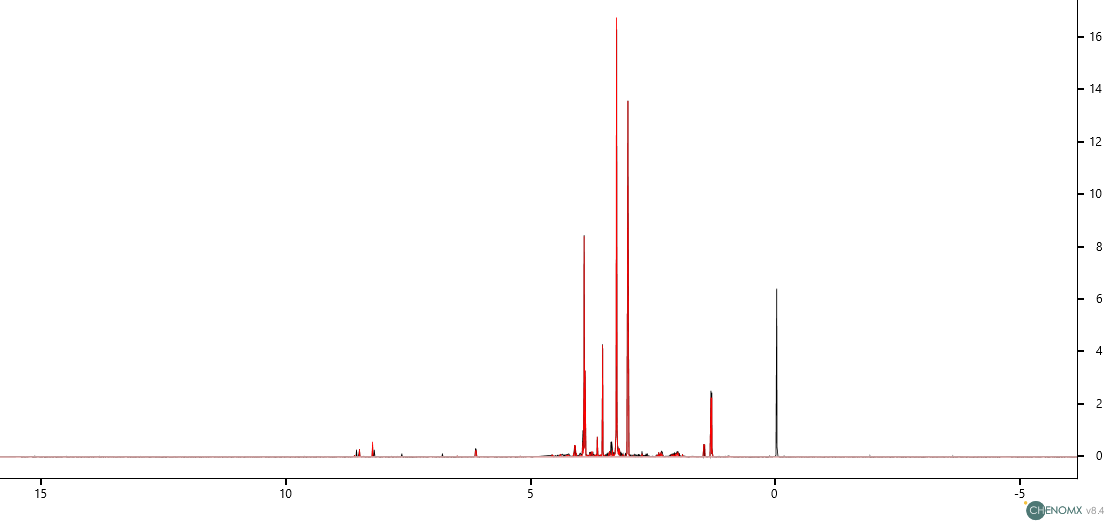

Supplement: Supplementary file 1 [file metabolites-13-00612-s001.zip › Figure_S1_NMR_spectrum_CTRL.png]

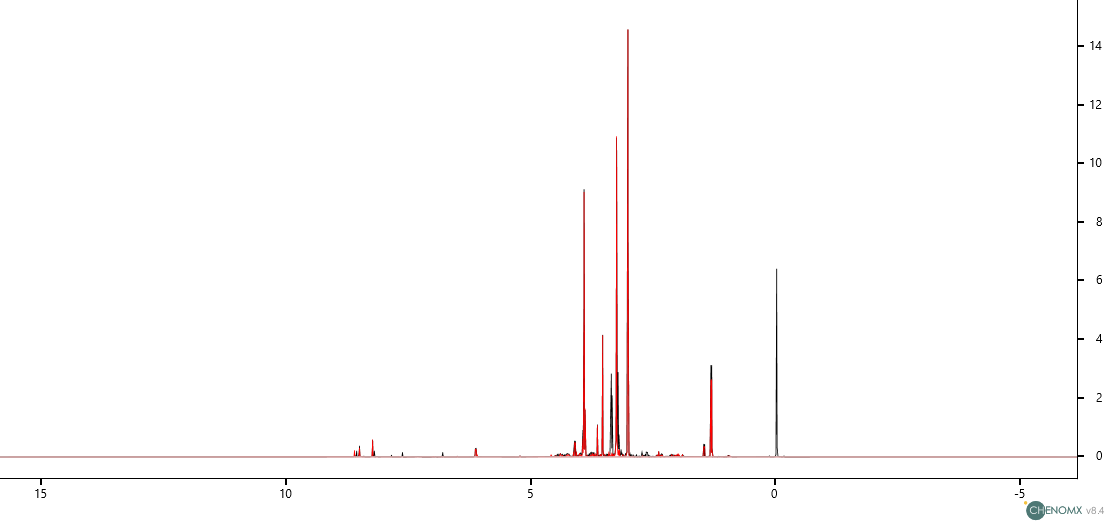

Supplement: Supplementary file 1 [file metabolites-13-00612-s001.zip › Figure_S2_NMR_spectrum_PLANT.png]

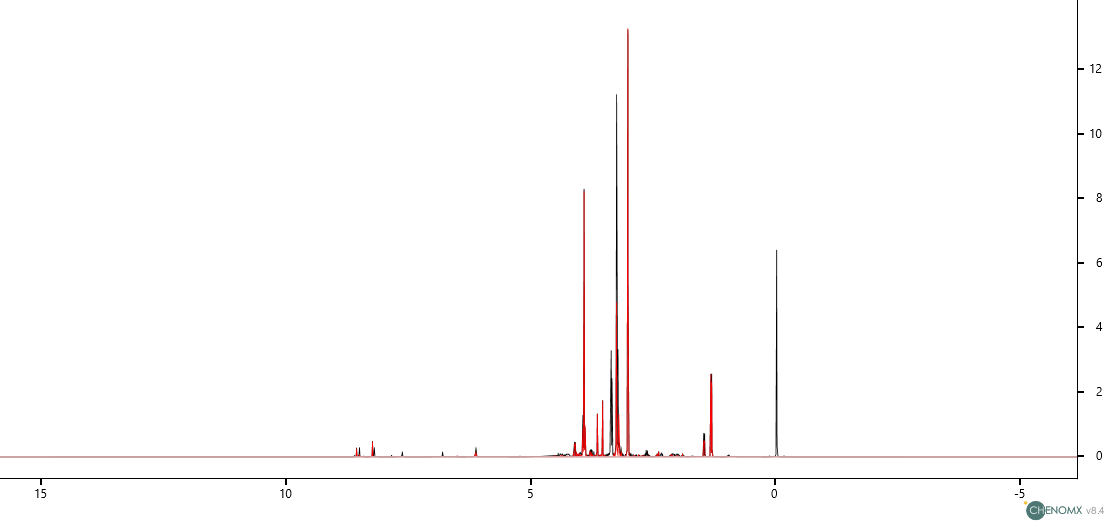

Supplement: Supplementary file 1 [file metabolites-13-00612-s001.zip › Figure_S3_NMR_spectrum_PAP.png]

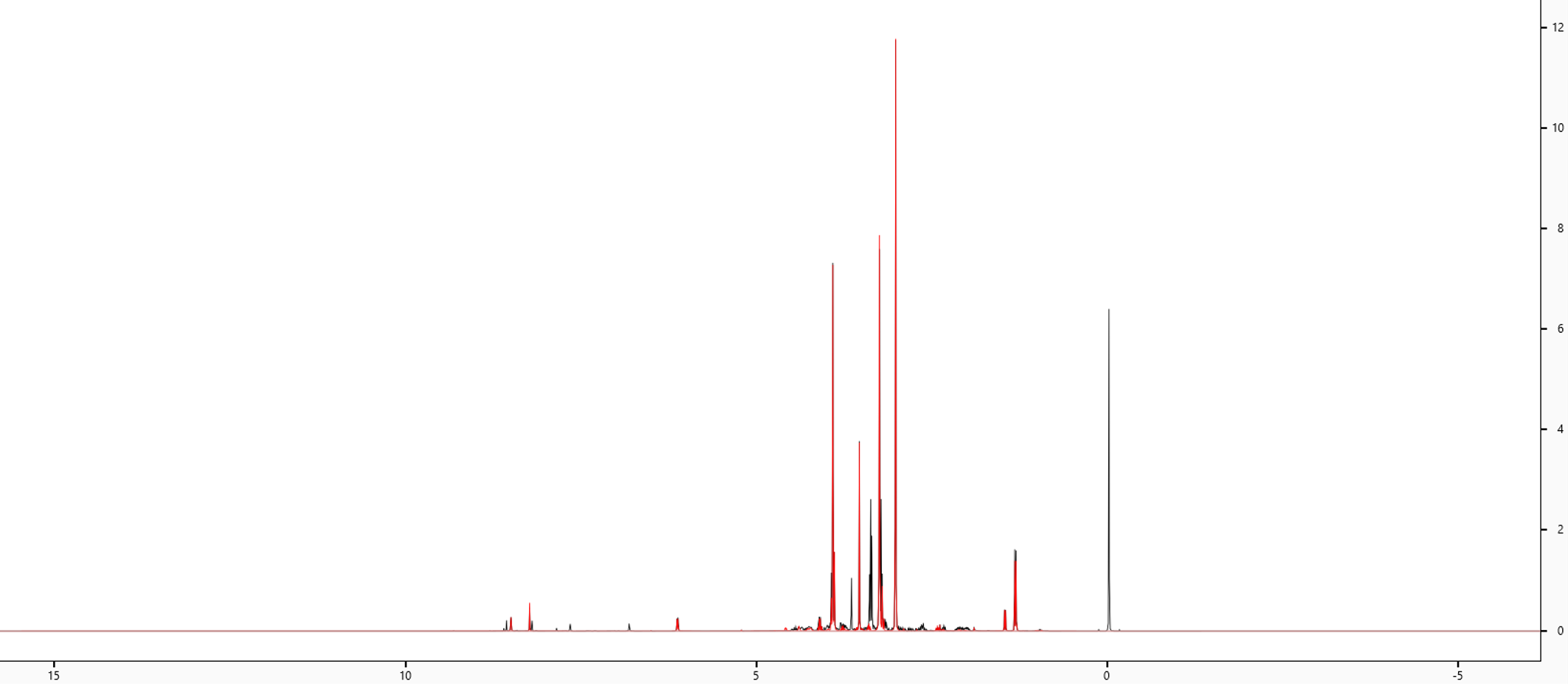

Supplement: Supplementary file 1 [file metabolites-13-00612-s001.zip › Figure_S4_NMR_spectrum_MIX.png]

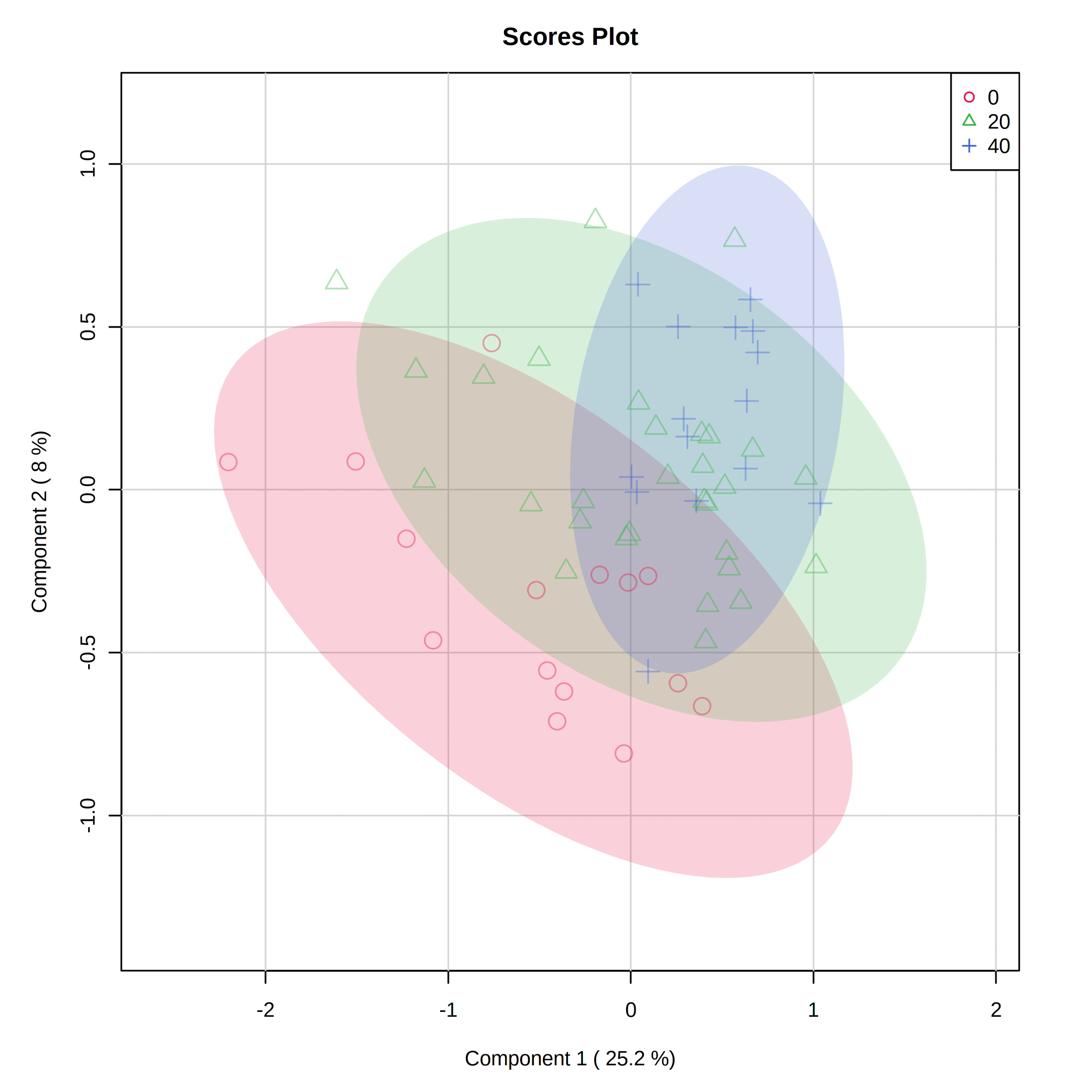

Supplement: Supplementary file 1 [file metabolites-13-00612-s001.zip › Figure_S5_PLS_DA_muscle_Fishmeal.png]
